# Synthesis, structure and blue-light emission of a zero-dimensional zinc halide with the 4-(4-chloro­phen­yl)pyridinium cation

**DOI:** 10.1107/S2053229625005200

**Published:** 2025-07-14

**Authors:** Ting Li, Hong-Ru Fu

**Affiliations:** a College of Chemistry and Chemical Engineering Luoyang Normal University,Luoyang 471934 People’s Republic of China; Oak Ridge National Laboratory, USA

**Keywords:** pyridinium, tetrabromidozincate, crystal structure, zinc hybrid halide, pyridine cation, blue emission

## Abstract

Exploring blue emission lead-free halides with high photoluminescence quan­tum yields (PLQYs) is a significant and challenging issue for luminescent materials. Herein, a zero-dimensional (0D) organic–inorganic hybrid halide, namely, bis­[4-(4-chloro­phen­yl)pyridinium] tetra­bromido­zincate, (C_11_H_9_ClN)_2_[ZnBr_4_] or [HCPP]_2_[ZnBr_4_] [CPP is 4-(4-chloro­phen­yl)pyridine], was synthesized through acid solution evaporation. The com­pound crystallizes in the monoclinic space group *C*2/*c*. The asymmetric unit consists of a [ZnBr_4_]^2−^ dianion cluster and two monoprotonated [HCPP]^+^ cations. The [ZnBr_4_]^2−^ unit presents an almost undistorted tetra­hedral geometry. The [HCPP]^+^ cations adopt nearly coplanar conformations with small dihedral angles of 5.05 (3)°; there are relatively strong π–π inter­actions between adjacent [HCPP]^+^ cations due to slipped parallel packing, with a distance of 3.81 (1) Å. Significantly, this halide exhibits highly efficient blue-light emission (385–500 nm) with a PLQY of 56.35%.

## Abstract 2

The hybrid halide [HCPP]_2_[ZnBr_4_] [HCPP is 4-(4-chloro­phen­yl)pyridinium] exhibits efficient blue emission centred at 432 nm with a photoluminescence quantum yield of 56.35%.

## Introduction

Recently, zero-dimensional (0D) metal hybrid halides have shown tremendous potential as luminescent materials due to their diverse structures and tunable photoluminescence (PL) (Han *et al.*, 2021 ▸; Haque *et al.*, 2025 ▸). Generally, 0D halides are com­posed of isolated metal halide clusters or units and bulky organic cations. In contrast to the high-dimensional halides, 0D hybrid halides always show strong quantum/dielectric confinement and possess much softer lattices, leading to an increased exciton binding energy, and allowing for the emission of self-trapped excitons (STEs) with wide broadband and large Stokes-shifted properties (Morad *et al.*, 2019 ▸; Dastidar *et al.*, 2024 ▸; Wang *et al.*, 2024*a* ▸). As a result, lower-energy green, yellow and red light emissions are easily realized in 0D halides, while higher-energy blue-light emission remains extremely challenging, especially in the pure-blue region (460–480 nm) (Ma *et al.*, 2021 ▸; Zhang *et al.*, 2021 ▸; Yang *et al.*, 2025 ▸). Great effort has been made to explore the preparation of blue-emitting 0D halides. For instance, Lei and co-workers have reported a series of 0D hybrid zinc halides of the form *A*ZnBr_4_ [*A* = 1-ethylpiperazine (EP), 1-phenylpiperazine (BP) and *N*,*N*,2,2-tetra­methyl­propane-1,3-di­amine (TMPDA)] based on discrete [ZnBr_4_]^2−^ tetra­hedra exhibiting blue emission (452–485 nm) and a maximum photolu­mi­nes­cence quan­tum yield (PLQY) of 35.47% (Ma *et al.*, 2022*a* ▸). Saparov and co-workers reported two halides, namely, (P-xd)ZnCl_4_ and (P-xd)CdCl_4_, where P-xd is *p*-xylylenedi­am­mo­nium, showing blue emission and a PLQY of 23% for (P-xd)ZnCl_4_ (Popy *et al.*, 2023 ▸). This group also utilized trimeth­yl(4-stilben­yl)methyl­ammonium (*R*^+^) as the organic cations to pre­pare indium(III) halides and the halide *R*InBr_4_ displays high-efficiency blue emission with a PLQY value of 16.36% (Fattal *et al.*, 2021 ▸). In addition, Zhang’s group prepared a 0D hybrid zinc halide, *i.e.* (C_9_H_14_N)_2_[ZnBr_4_] (C_9_H_14_N^+^ is the *N*-butyl­pyridinium cation) based on the pyridine quaternary ammonium salt. This crystal shows efficient blue-light emission centred at 426 nm and a high quantum yield of 49.95% (Cheng *et al.*, 2023 ▸). Despite the significant achievements that have been made in the development of 0D lead-free halides, achieving highly efficient blue-light emission is extremely challenging due to the limitation of an intrinsic large Stokes shift and an unavoidable self-absorption effect (Ma *et al.*, 2022*b* ▸; Peng *et al.*, 2021 ▸; Cho *et al.*, 2025 ▸). However, after summarizing the reported studies about blue-emitting 0D halides, we found that the construction of blue-emitting 0D halides is mainly concentrated on two strategies. The first common strategy is to adopt *d*^10^ metal ions, such as Zn^2+^ and Cd^2+^, which have a relatively stable outer electronic structure (Liu *et al.*, 2024 ▸; Tan *et al.*, 2025 ▸; Hao *et al.*, 2022 ▸). The other is to focus on the modulation of organic cations. The organic com­ponents play a crucial role in generating blue emission and the introduction of an organic cation into a hybrid structure can improve its blue-emission efficiency (Deng *et al.*, 2023 ▸; Belikova *et al.*, 2024 ▸). These findings provide important inspiration for the design of hybrid halides as blue-light emitters.
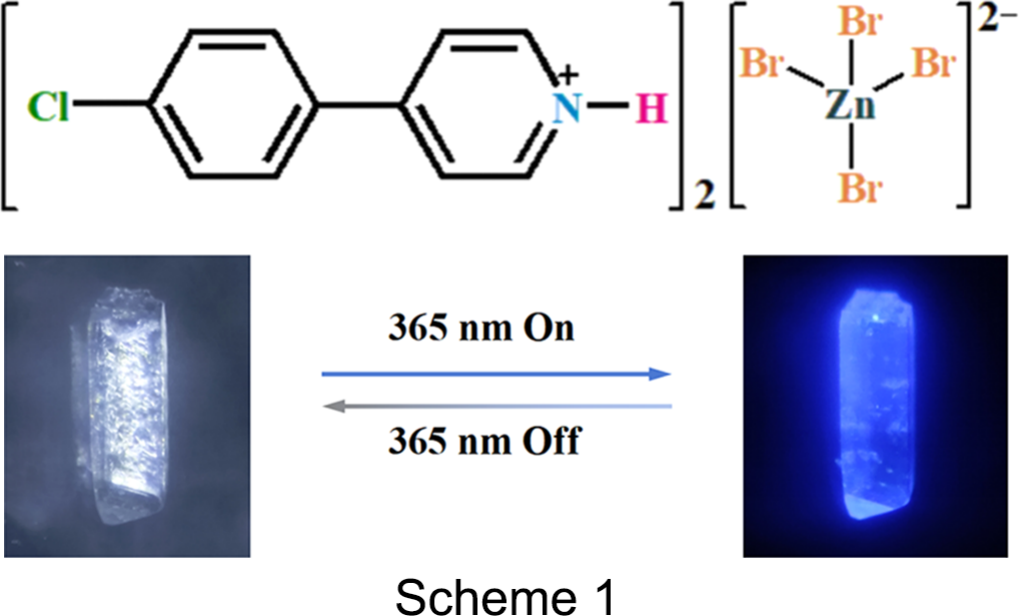


In this study, a new 0D organic–inorganic hybrid halide, namely, bis­[4-(4-chloro­phen­yl)pyridinium] tetra­bromido­zinc­ate, [HCPP]_2_[ZnBr_4_], was prepared by adopting chlorine-de­cor­ated 4-phenyl­pyridine as the cation source. This halide exhibits an intense blue emission centred at 432 nm through STEs of the [ZnBr_4_]^2−^ unit and a singlet excited state of the organic cations with a PLQY of 56.35%, surpassing almost all previously reported 0D zinc halide counterparts. This work provides an efficient way to achieve high-efficiency blue emission through the modulation of appropriate organics.

## Experimental

### Materials and methods

Zinc bromide (anhydrous, ZnBr_2_, 99.9%) and 4-(4-chloro­phen­yl)pyridine (CPP, 99.0%) were purchased from Alfa, and ethanol (EtOH, 99.9%) and hy­dro­gen bromide (HBr, 35%) were purchased from Aladdin. All chemicals are used directly without further purification. Elemental analysis for C, N and H was performed on an Elementar Vario MICRO instrument. Powder X-ray diffraction (PXRD) analysis was carried out with a Rigaku D/Max 2500 powder X-ray diffractometer using Cu *K*α radiation (λ = 1.5418 Å) at a voltage of 40 kV and 40 mA. Photoluminescence (PL) spectra, quantum yield and time-resolved decay spectra were recorded on an Edinburgh FLS1000 fluorescence spectrometer with a xenon lamp and an integrated sphere sample chamber.

### Synthesis and crystallization

For the synthesis of [HCPP]_2_[ZnBr_4_], a mixture of CPP (0.2 mmol) and ZnBr_2_ (0.1 mmol) was dissolved in a mixture of ethanol (3 ml) and HBr (1 ml), and stirred at 353 K for 30 min. Colourless block-shaped crystals suitable for single-crystal X-ray diffraction analysis were obtained by slow evaporation under ambient conditions for 1 d. The crystals were collected, washed with acetone five times and then dried in air (yield 63.5%, based on Zn). Analysis calculated (%) for C_22_H_18_Br_4_Cl_2_N_2_Zn: C 31.58, H 2.35, N 3.13; found: C 32.04, H 2.38, N 3.19.

### Refinement

Crystal data, data collection and structure refinement details are summarized in Table 1 ▸. H atoms connected to C atoms were located at geometrically calculated positions.

### Density Functional Theory (DFT) calculations

First-principles calculations were carried out using the projected augmented-wave (PAW) method (Kresse *et al.*, 1996 ▸), as implemented in *VASP* (Kresse *et al.*, 1999 ▸; Perdew *et al.*, 1992 ▸). The exchange correlation energy was treated using the Perdew–Burke–Ernzerhof (PBE) exchange–correlation functional in the scheme of generalized gradient approximation (Hohenberg *et al.*, 1964 ▸; Perdew *et al.*, 1996 ▸). The kinetic energy cutoff for all cases was determined to be 520 eV. The convergence thresholds for the electronic calculations and ionic relaxations were chosen as 10^−6^ eV and 0.01 eV Å^−1^, respectively. The standard Monkhorst–Pack k-point grids with a density of 0.1 Å^−1^ were used for Brillouin zone sampling. The valence electron configurations applied in this work were treated as Zn 3*d*^10^4*s*^2^, C 2*s*^2^2*p*^2^, Cl 3*s*^2^3*p*^5^, Br 4*s*^2^4*p*^5^, N 2*s*^2^2*p*^3^ and H 1*s*^1^.

## Results and discussion

**Figure 1:**
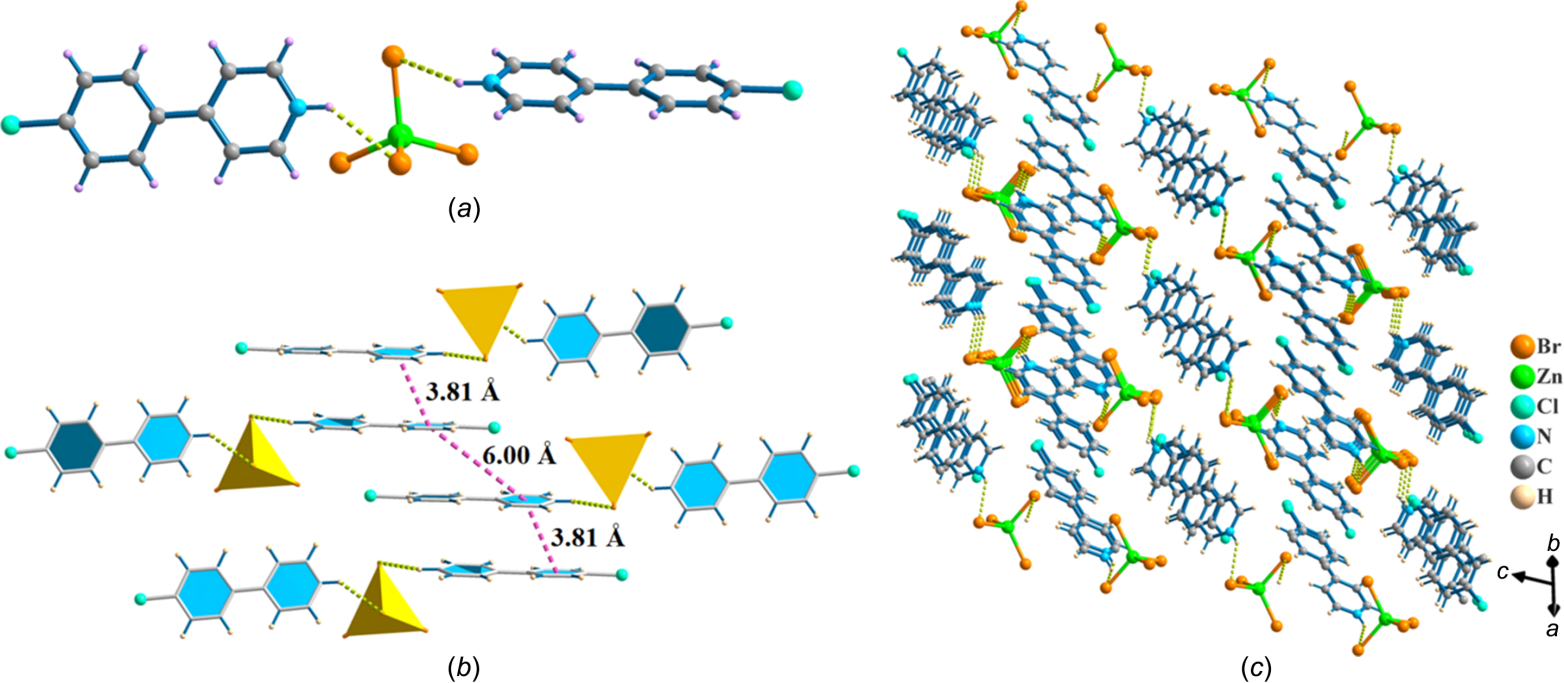
The mol­ecular structure of [HCPP]_2_[ZnBr_4_], showing (*a*) the tetra­hedral [ZnBr_4_]^2−^ anion and [HCPP]^+^ cation, (*b*) the inter­actions between the cations and inorganic nodes, and (*c*) a packing diagram of [HCPP]_2_[ZnBr_4_].

Single-crystal X-ray diffraction analysis reveals that [HCPP]_2_[ZnBr_4_] crystallized in the monoclinic space group *C*2/*c*. The asymmetric unit consists of two [HCPP]^+^ cations and one [ZnBr_4_]^2−^ anion. As shown in Fig. 1 ▸(*a*), each Zn^2+^ ion is coordinated to four Br^−^ anions to form a [ZnBr_4_]^2−^ tetra­hedron, yielding a zero-dimensional (0D) structure, with an average Zn—Br bond length of 2.4055 (4) Å and an average Cl—Zn—Cl bond angle of 109.04 (3)°, which are close to those of [ZnBr_4_]^2−^ tetra­hedra in previously reported Zn-based hybrid halides (He *et al.*, 2023 ▸). Each [ZnBr_4_]^2−^ tetra­hedron connects two [HCPP]^+^ cations *via* inter­molecular hy­dro­gen bonds to form a 0D dimer; the Br⋯H—N angle is 140.47 (3)°. One [HCPP]^+^ cation of this dimer is arranged parallel to one [HCPP]^+^ cation of another dimer, with an inter­planar distance of 3.81 (1) Å [Fig. 1 ▸(*b*)]. These dimers are further stacked into a 3D supra­molecular structure [Fig. 1 ▸(*c*)]. Notably, the pyridine and arene rings are nearly coplanar in the [HCPP]^+^ cation, with a small dihedral angle between their planes of 5.05 (3)°. The degree of structural distortion of the ZnBr_4_ tetra­hedron was evaluated by calculating the distortion of the bond lengths (Δ*d*) according to the equation of Robinson *et al.* (1971 ▸). The calculated Δ*d* value is 1.14 × 10^−4^ and such a small degree of distortion (Liu *et al.*, 2023 ▸; Wang *et al.*, 2024*b* ▸) suggests the weak STEs of the [ZnBr_4_]^2−^ cluster, which is good for the generation of the narrow emission band of [HCPP]_2_[ZnBr_4_].

**Figure 2:**
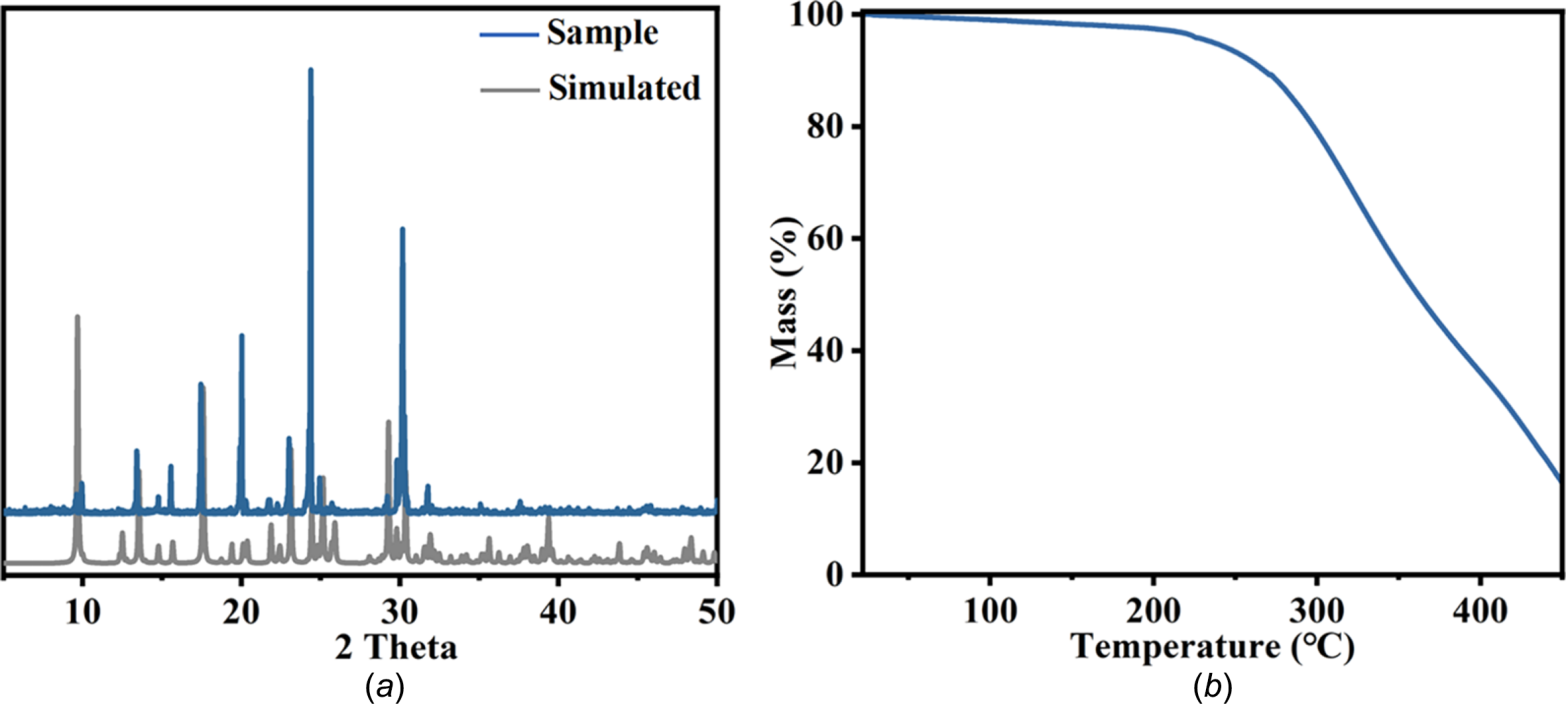
The basic characterization of [HCPP]_2_[ZnBr_4_], showing (*a*) experimental and simulated PXRD patterns of [HCPP]_2_[ZnBr_4_] and (*b*) thermal analysis of [HCPP]_2_[ZnBr_4_].

The powder X-ray diffraction (PXRD) pattern of [HCPP]_2_[ZnBr_4_] matches well with that simulated from the obtained single-crystal data without any extra diffraction peaks, indicating their high purity [Fig. 2 ▸(*a*)]. The thermogravimetric (TG) curve shows that the [HCPP]_2_[ZnBr_4_] crystals have excellent thermal stability; they begin to decom­pose from ∼250 °C [Fig. 2 ▸(*b*)].

**Figure 3:**
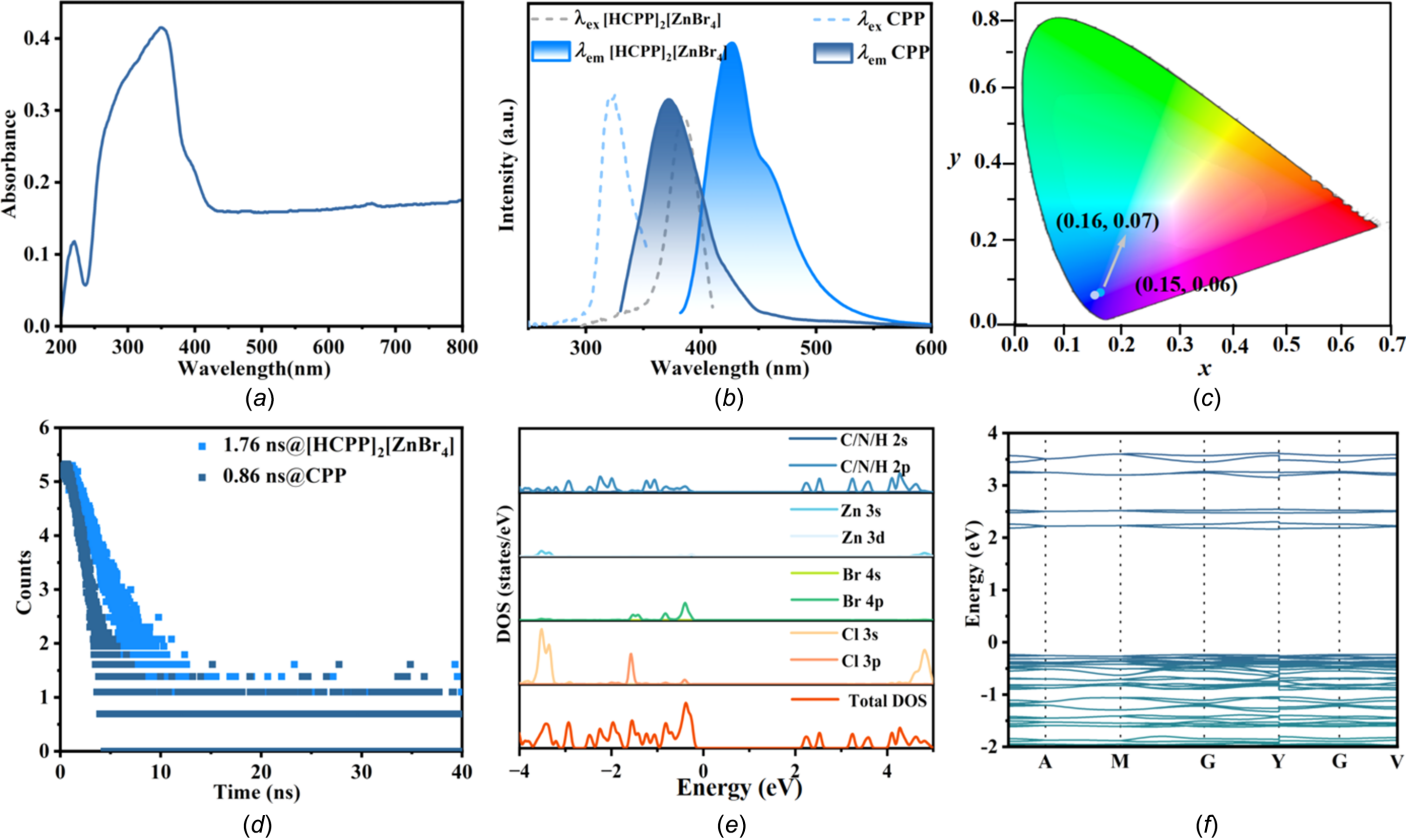
The luminescence properties and theoretical calculations for [HCPP]_2_[ZnBr_4_], showing (*a*) the solid-state UV–Vis absorption spectrum of [HCPP]_2_[ZnBr_4_], (*b*) the excitation and emission spectra of organic mol­ecule CPP and [HCPP]_2_[ZnBr_4_], (*c*) the CIE chromaticity coordinates, (*d*) the PL decay curves of CPP and [HCPP]_2_[ZnBr_4_], (*e*) DOS and (*f*) calculated band structure.

The photophysical properties of [HCPP]_2_[ZnBr_4_] were investigated. As shown in Fig. 3 ▸(*a*), the solid-state UV–Vis absorption spectrum of [HCPP]_2_[ZnBr_4_] shows that the band edge is at about 400 nm. Upon 360 nm excitation, [HCPP]_2_[ZnBr_4_] exhibits a narrow blue-emission band centred at 432 nm, with the full-width at half-maximum (FWHM) of 76 nm, a large Stokes shift of 46 nm and a high PLQY of 56.35%. The Commission Inter­nationale de l’Éclairage (CIE 1931) chromaticity coordinates were determined as (0.16, 0.07) [Fig. 3 ▸(*c*)]. The PL decay lifetime is 1.76 ns at 432 nm [Fig. 3 ▸(*d*)].

In addition, the luminescence behaviour of CPP powder was tested and it exhibited a weak blue emission with a peak at 375 nm under 320 nm excitation [Fig. 3 ▸(*b*)], a PLQY of 11.36% and the CIE chromaticity coordinates (0.15, 0.06). The time-resolved spectrum of CPP under ambient conditions revealed a delay time of 0.86 ns at 375 nm [Fig. 3 ▸(*d*)]. Compared to the PLQY of CPP, the PLQY of [HCPP]_2_[ZnBr_4_] shows a 4.96-fold improvement, which may be attributed largely to the ordered arrangement and rigid enhancement of CPP mol­ecules in the halide matrix, leading to nonradiative transition suppression and PLQY improvements.

To better understand the luminescence mechanism of [HCPP]_2_[ZnBr_4_], DFT calculations were performed to investigate the band structure and density of states (DOS). The valence band maximum (VBM) of [HCPP]_2_[ZnBr_4_] is mainly located in the [HCPP]^+^ cation and the Br 4*p* orbital, and the conduction band maximum (CBM) is mainly distributed in the [HCPP]^+^ cation [Fig. 3 ▸(*e*)]. The energy band gap between the valence and conduction band of [HCPP]_2_[ZnBr_4_] is 2.48 eV [Fig. 3 ▸(*f*)], with such a small value indicating an intense quantum confinement effect. These results further confirm that the enhancement of blue emission of [HCPP]_2_[ZnBr_4_] originates from the organic cations and the STE of the [ZnBr_4_]^2−^ node (Qi *et al.*, 2022 ▸).

## Summary

By adopting chlorine-decorated 4-phenyl­pyridine, a zinc-based hybrid halide has been prepared. It exhibits efficient blue emission with a high PLQY of 56.35%, among the highest reported in the literature to date. Experimental and theoretical studies indicate that the emission originates from the synergistic function of the electron transitions in the organic cations and the STE states. This work lays the foundation for synthesizing more efficient blue-emission hybrid halides.

## Supplementary Material

Crystal structure: contains datablock(s) I, global. DOI: 10.1107/S2053229625005200/vx3015sup1.cif

Structure factors: contains datablock(s) I. DOI: 10.1107/S2053229625005200/vx3015Isup2.hkl

CCDC reference: 2452243

## Figures and Tables

**Table 1:** Experimental details

Crystal data	
Chemical formula	(C_11_H_9_ClN)_2_[ZnBr_4_]
*M*_r_	766.27
Crystal system, space group	Monoclinic, *C*2/*c*
Temperature (K)	285
*a*, *b*, *c* (Å)	12.7583 (6), 14.1507 (5), 15.2706 (5)
β (°)	110.067 (1)
*V* (Å^3^)	2589.57 (18)
*Z*	4
Radiation type	Mo *K*α
μ (mm^−1^)	7.34
Crystal size (mm)	0.16 × 0.16 × 0.15

Data collection	
Diffractometer	Bruker APEXII CCD
Absorption correction	Multi-scan (*SADABS*; Bruker, 2016 ▸)
*T*_min_, *T*_max_	0.321, 0.333
No. of measured, independent and observed [*I* > 2σ(*I*)] reflections	42246, 3223, 2597
*R*_int_	0.074
(sin θ/λ)_max_ (Å^−1^)	0.667

Refinement	
*R*[*F*^2^ > 2σ(*F*^2^)], *wR*(*F*^2^), *S*	0.027, 0.067, 1.02
No. of reflections	3223
No. of parameters	145
H-atom treatment	H atoms treated by a mixture of independent and constrained refinement
Δρ_max_, Δρ_min_ (e Å^−3^)	0.26, −0.61

Computer programs: *APEX2* (Bruker, 2016 ▸), *SAINT* (Bruker, 2016 ▸), *SHELXT* (Sheldrick, 2015*a* ▸), *SHELXL2014* (Sheldrick, 2015*b* ▸) and *OLEX2* (Dolomanov *et al.*, 2009 ▸).
